# The value of leading customers in a crowdfunding-based marketing pattern

**DOI:** 10.1371/journal.pone.0215323

**Published:** 2019-04-15

**Authors:** Jingjing Zhao, Yongli Li, Yunlong Ding, Chao Liu

**Affiliations:** 1 School of Management, Harbin Institute of Technology, Harbin, P.R. China; 2 School of Business Administration, Northeastern University, Shenyang, P.R. China; Shandong University of Science and Technology, CHINA

## Abstract

Crowdfunding is gradually becoming a modern marketing pattern. By noting that the success of crowdfunding depends on network externalities, our research aims to utilize these network externalities to provide an applicable referral mechanism in a crowdfunding-based marketing pattern. In the context of network externalities, measuring the value of leading customers is chosen as the key to coping with the research problem, as the leading customers are considered to be critical in forming a referral network. Accordingly, two sequential-move game models (i.e., the basic model and the extended model) were established to measure the value of leading customers, and a matrix transformation process was adopted to solve the model by transforming a complicated multisequence game into a simple simultaneous-move game. Based on the defined value of leading customers, a network-based referral mechanism was proposed that explores exactly how many awards should be allocated along the customer sequence to encourage the leading customers to provide a successful recommendation, and this demonstrates two general rules of awarding the referrals in our model setting. Moreover, the proposed solution approach helps to deepen the understanding of the effect of the leading position, which is meaningful for designing a greater number of referral approaches.

## Introduction

With the widespread phenomenon of the sharing economy, crowdfunding is gradually moving beyond its traditional functions, such as funding artistic or creative projects and is becoming a modern marketing mode, with the aim of selling various types of products or services, typically through online platforms [1]. For example, several E-commerce platforms, such as JD (https://z.jd.com/sceneIndex.html) and Taobao (https://izhongchou.taobao.com/index.htm), contain a crowdfunding-based marketing model that is being used to market numerous categories of products and services, such as clothes, fruit, wine, electronic products and even household services. Regarding this new marketing mode, the core problem we consider here is how to provide several marketing strategies in general, and, in particular, how to provide an applicable referral mechanism for crowdfunding-based marketing models.

Generally, the crowdfunding process consists of three elements: the initiator, who proposes the idea or project to be funded; the individuals or customers, who support the idea; and a platform, which brings the two parties together to conduct the crowdfunding [2]. If crowdfunding is understood to be a new marketing tool, it possesses several features. First, the crowdfunding initiator acts both as the sole producer and the seller of the crowdfunding project, which is different from the traditional supply chain, where the producer and the seller are always in different sections of the supply chain. Second, crowdfunding has the function of market discovery; for example, if the crowdfunding campaign is successful, it means that the targeted product or service is welcomed by the customers, and the initiator can determine in advance how many customers will buy the products or services, how much they will buy and who they are; if the crowdfunding is not successful by the deadline, the initiator can infer that the targeted product or service is not accepted by the market; thus, the initiator does not need to produce or offer the good or the service. However, the traditional supply chain cannot perform as well as the crowdfunding supply chain because the traditional producer or seller cannot know in advance whether the offered products or services will be accepted by customers or the extent of acceptance. Third, with the development of information technology, modern crowdfunding is much more dependent on online platforms; thus, the word-of-mouth communication captured by local network externalities or the social learning embedded in social networks cannot be ignored, especially when potential consumers make their consumption decision and crowdfunding initiators offer their pricing and marketing strategies [3].

Accordingly, when we study and explore the referral mechanism for the growth of crowdfunding-based marketing models, we should pay close attention to the abovementioned three features. Specifically, in response to the first feature, we need only consider two types of actors in our model—the initiator and the customers—because the crowdfunding initiator acts as both the producer and seller. Furthermore, the initiator can be regarded as the monopolist within the launched crowdfunding project, so that the established model features the monopoly structure. Please note that, although many crowdfunding projects can be launched almost at the same time via online platforms, the substitutability between these projects is always very low because of the originality that crowdfunding projects often offer. Thus, one crowdfunding campaign can be understood as a monopoly market for the targeted project, especially during the launch period, which is often not long. Next, with regard to the second feature, the information known in advance can facilitate the growth of participating customers (or, say, the market size), the customer sequence (or, say, the order of each customer who participates in the crowdfunding campaign) and even the relationships of those who successfully recommend others to participate in the crowdfunding campaign. Finally, network externalities should be added into the analysis according to our above statements. In total, the monopoly structure, customer sequence and network externalities are the three critical considerations in the process of solving the proposed research problem.

Since the monopoly structure has been discussed widely in the literature related to Economics and Management, we first focus on analyzing the other two considerations, i.e., network externalities and the customer sequence. Here, we will focus on how to reflect and to depict them in our model; further, we provide some hypotheses about their potential relationships with possible market strategies. Numerous researchers, such as Anderson et al. [4] and Wang and Wang [5], have recently considered and studied network externalities in online E-commerce platforms, especially in the context of the monopoly structure, such as Crapis et al. [6], Ajorlou et al. [7] and Shin [8]. Based on these studies, utilizing network externalities to improve marketing effectiveness is a feasible idea [9]. For example, we can find the important nodes with highly positive network externalities and design mechanisms that allow these nodes to participate in enlarging markets and increasing profits [10]. Following this idea, we consider network externalities and their effects on the customers’ consumption decisions, i.e., the utility derived by a consumer from consuming a targeted good or service is significantly affected by his/her network peers who also consume that good or service. With regards to the customer sequence, it is a common phenomenon that some customers join a crowdfunding project earlier than others. The earlier customers can potentially recommend the good or the service to their social network peers in a social media platform, thus, these earlier customers are able to affect the marketing effects or even the profits earned by the crowdfunding initiator (or the monopolist, hereafter). In such a case, the earlier customers are called the leading customers (or leaders, hereafter), and the latter ones are called the following customers (or followers, hereafter). In fact, the idea of the Von Stackelberg [11] game can be adopted in our model to reflect the mutual effects between leaders and followers.

Based on the three features of crowdfunding, the core idea of solving the research problem is to explore and measure the value of leading customers in the context of network externalities. Here, the value of leading customers is measured by the increased profits gained by the monopolist when some customers become the leaders from the known customer set. Once the value of the leading customers is determined, we can calculate how much profit can be returned or awarded to the leading customers to incentivize them to recommend more new customers. Therefore, the likelihood of successfully conducting a crowdfunding project can be increased. However, different customers can be located at different time points in the formed customer sequence, which means that some customers can be their earlier ones’ followers and their later ones’ leaders. Accordingly, one challenging problem is how to quantitatively allocate the awards to different customers with different time stamps along the customer sequence. Fortunately, the idea of measuring the value of the leading customer can also be adopted in this complicated case, where the contributions of different customers to the increased profits earned by the monopolist can be similarly determined. Accordingly, once the different contributions can be determined, the quantitative model of encouraging the leading customers to increase their recommendations can be proposed. In other words, the proposed approach of allocating the increased profits along the customer sequence can be adopted to award the action of successfully recommending new customers.

The remaining part of this section first reviews the related models in this field and then identifies our contributions. The following sections are organized as follows: Section 2 provides the sequential-move game model that highlights the following three features: the monopoly structure, network externalities and customer sequence; Section 3 presents the established model’s consumption equilibrium and its optimal pricing strategy; Section 4 studies the relationship between our sequential-move game model and the known simultaneous-move game by way of matrix transformations and further discusses how to understand the influence of leading positions in the sequential-move game; Section 5 utilizes the results reported in Section 4 and focuses on measuring and illustrating the value of leading customers; Section 6 shows the application of our models and provides answers to the research problem; and finally, Section 7 concludes.

### Related work

Aside from the abovementioned background of crowdfunding, our study is also related to the scientific problem of how to design a customer utility function to reflect the local network externality. Fortunately, Ballester et al. [12] established a quadratic utility function that can reflect the peer effect of the local network externality, and then Candogan et al. [13] and Bloch and Querou [14] extended this utility function by adding the prices offered by the monopolist into the utility function. Because the designed quadratic utility function facilitates the mathematical analysis and is also able to reflect the law of diminishing marginal utility, it has been widely adopted in recent years by, for example, by Zhou and Chen [15], Li et al. [16], Fainmesser and Galeotti [17] and Zhou and Chen [18]. As mentioned above, crowdfunding features a monopolistic structure and network externalities, thus, the work by Candogan et al. [13] and Bloch and Querou [14] inspire the current study’s approach to designing the utility function with local network externalities. Accordingly, this paper inherits the customer’s utility functions designed by Candogan et al. [13] and Bloch and Querou [14].

Another important factor addressed here is the leading customer in the customer sequence, and we want to explore how the leader affects the followers’ decisions and how the leading position affects the initiator’s profits. Once the patterns of influence can be uncovered, we can provide marketing strategies by utilizing the leading customer’s effects [19,20]. In fact, starting from the Von Stackelberg [11] game model, the effect of the leading customer has been considered by an increasing number of researchers. For example, Markides and Sosa [21] discussed the advantages and disadvantages of leading positions, and Klingebiel and Joseph [22] revealed how firms make decisions on becoming the leading mover in innovation in the case of mobile phone technologies. In the context of local network externalities, Zhou and Chen [15] adopted the utility function designed by Ballester et al. [12], analyzed a two-stage game and then provided the approach to finding the key players in social networks. However, the work of Zhou and Chen [15] does not consider the effect of the monopoly or the producers and sellers in the real market, in general, but their idea is important for us in modeling the customer sequence. From this perspective, our work can be regarded as extending that of Zhou and Chen [15] into a real market situation containing not only customers but also producers and sellers. In addition, our paper focuses on measuring the value of leading customers and aims to provide an applicable referral mechanism; thus, it is different from Zhou and Chen [15] and their extended work [18].

### Contributions

As crowdfunding is a relatively new phenomenon and a gradual marketing mode, the literature on crowdfunding has been growing. Although these previous studies discuss many important problems in crowdfunding, such as the optimal pricing mechanism [23], the success likelihood [24] and many others not mentioned, few studies focus on how to introduce the referral mechanism into a crowdfunding-based market model, let alone the utilization of the network externalities to provide a network-based referral mechanism. More specifically, this paper not only analyzes three features of modern crowdfunding—monopoly structure, network externalities and customer sequence—but it also models them to offer several new management insights into how to increase the crowdfunding initiator’s profits and how to raise the success likelihood of crowdfunding.

Second, this paper supplemented the theories of customer power by revealing a new customer power, which is called the leading effect, which can be understood as a type of demonstration effect. Traditionally, customer power is often understood as the ability of a customer to influence the decisions of a manufacturer [25,26] and is divided into the following five sources: expert power, referent power, legitimate power, reward power and coercive power [27]. However, the measured value of the leading customer in this paper can be regarded as another customer power because the measured value can tell who can cause a larger profit increase when they become the leading customers. Accordingly, the contribution of uncovering another customer power will benefit the design of a network-based referral mechanism.

Note that one recent work [18] that studied optimal pricing with sequential consumption in networks found that the optimal sequence should be a chain structure. Although our model setting is quite similar to the cited work, our research aim is quite different. First, our work focuses on measuring the value of leading customers by solving the sequential-move game and further proposing an applicable network-based referral mechanism; however, the work of Zhou and Chen [18] focused on finding the key players in a sequential-move game, uncovering the optimal sequence structure that causes the largest profit of the monopolist as well as comparing the different effects of symmetric and asymmetric social interactions on the pricing mechanism and the achieved profits. Second, although a similar solution technique was adopted in our work and that of Zhou and Chen [18], our study focused on adopting the solution technique to link the sequential-move game with the equivalent simultaneous-move game and further deepening our understanding the effect of leading positions, whereas the work of Zhou and Chen [18] did not focus on such problems. These differences, in our opinion, constitute one of our contributions. In fact, when we began this work and had finished most of it, the work of Zhou and Chen [18] had not been published publicly in SSRN; thus, although some of the results in this paper, such as Lemma 2, are consistent with the work of Zhou and Chen [18], the proof processes are quite different. However, despite these differences and although the main contributions are different, we cannot deny that the works of Candogan et al. [13], Zhou and Chen [15] and Zhou and Chen [18] are foundational in this field and provide the important idea and basic framework for our study.

## Model setup

### Utility function with a referral network

As we have explained, the crowdfunding-based marketing model consists of one monopolist who provides a divisible good and a finite set *N* = {1,2,⋯,*n*} of consumers embedded in an information network that reflects the local network externality. Here, let the adjacency matrix ***IN*** represent the information network, and if customers *i* and *j* can share information with each other, then the *ij*-entry and *ji*-entry of the matrix ***IN*** are assigned as 1; otherwise, they are assigned as 0. Based on the real existing network ***IN***, we further introduce and design a referral network ***R*** into the crowdfunding-based marketing pattern. Similar to the information network ***IN***, the *ij*-entry of ***R*,** denoted as *r*_*ij*_, also takes two values, as follows: *r*_*ij*_ = 1, which means customer *j* successfully recommend customer *i* to participate and *r*_*ij*_ = 0, which customer *j* is not successful in recommending customer *i* to participate. Accordingly, *IN*_*ij*_ = 1 or *IN*_*ji*_ = 1 is the sufficient condition of *r*_*ij*_ = 1 by considering that sharing information is the precondition of a recommendation; additionally, the referral network ***R*** is direct, because the action of a successful recommendation is naturally direct, whereas the information network ***IN*** is undirected. The established model will focus on the referral network ***g*** with the aim of determining how it influences the monopolist’s profit and how to design it to increase the monopolist’s profit.

Following the traditional utility form adopted in Candogan et al. [13] and Bloch and Quérou [14] as mentioned in the Related work section, we inherit the utility function of the quadratic form, but we focus on the effect of our introduced referral network. In other words, although the network externality can originate from many aspects and takes many forms, this paper focuses on the effect of referral network. In this paper, we pay attention to ***two types of effects*** from the introduced referral network on the customer’ utility: one is the influence from the referrers and the other comes from the successfully recommended customers. In other words, the utility of customer *i* is not only affected by the customers who recommend the good to him/her but is also affected by the customers who successfully accept his/her recommendation, and further, we allow the different influence strengths from the two directions. Let *η* represent the different strengths, where *η*>1 means the latter effect is stronger than the former and 1>*η*>0 means the opposite. Accordingly, the specific utility function of customer *i* is designed as follows:

$$u_{i}(x_{i};x_{-i},p_{i})=\underset{\mathrm{effect}\;\mathrm{from}\;\mathrm{individual}\;\mathrm{preference}}{\underset{︸}{\alpha_{i}x_{i}-\frac{1}{2}\beta_{i}x_{i}^{2}}}+\underset{\mathrm{influence}\;\mathrm{from}\;\mathrm{referrers}}{\underset{︸}{\left(\sum_{j=1}^{n}r_{ij}x_{j}\right)x_{i}}}+\underset{\begin{array}{c} \mathrm{influence}\;\mathrm{from}\;\mathrm{the}\;\mathrm{successfully}\\ \mathrm{recommended}\;\mathrm{customers} \end{array}}{\underset{︸}{\left(\eta \sum_{j=1}^{n}r_{ji}x_{j}\right)x_{i}}}\;\underset{\mathrm{purchase}\;\mathrm{cost}}{\underset{︸}{-p_{i}x_{i}}},$$ (1A)

where *x*_*i*_ is the amount that consumer *i* decides to purchase, **x**_−*i*_ is the consumption vector excluding consumer *i*, and *p*_*i*_ is the price offered to consumer *i* by the monopolist for one unit of the divisible good. Aside from the explained two effects in the middle of Eq (1A), two other important effects are also considered in the designed utility function. For the effect from individual preference, *α*_*i*_ and *β*_*i*_ together represent the preference level of consumer *i* for the good; furthermore, *α*_*i*_ measures the added utility by consuming one more unit of the good, and *β*_*i*_ reflects the law of decreasing margin utility. For the purchase cost, the last term measures the cost to consumer *i* of purchasing *x*_*i*_ units of the good; in addition, the offer price *p*_*i*_ can be different for different customers, noting that the monopolist enables price discrimination for different consumers according to their various purchasing times as well as their positions in the referral network.

To facilitate expressing the results, we add the two effects of the referral network together to establish a new comprehensive network **g,** of which the *ij*-entry is expressed as follows:

$$g_{ij}=r_{ij}+\eta \cdot r_{ji},$$ (1B)

and then, the designed utility function in Eq (1A) can be simplified as follows:

$$u_{i}(x_{i};x_{-i},p_{i})=\alpha_{i}x_{i}-\frac{1}{2}\beta_{i}x_{i}^{2}+\left(\sum_{j=1}^{n}g_{ij}x_{j}\right)x_{i}-p_{i}x_{i}$$ (1C)

whose form is identical to those of Candogan et al. [13] and Bloch and Quérou [14]; however, the implication of the contained network is completely different. Moreover, the complicated form shown in Eq (1A) will also be useful in uncovering the implications of leading positions as well as to comparing the two effects (i.e., influence from referrers and influence from the successfully recommended customers) on increasing the monopolist’s profit.

### Profit function with differential pricing

In our model set, the monopolist is allowed to conduct complete price discrimination; therefore, customers can be offered different prices according to their preferences, purchasing sequence and network positions. Although obstacles can exist to inhibit complete price discrimination in real markets, this assumption will also be useful in providing several foundational theoretical results. Next, the same production cost *c* is assumed for each unit of the good, and then, the monopolist faces the following profit function:

$$\pi (x_{i},p_{i})=\sum_{i=1}^{n}(p_{i}-c)x_{i}$$ (2)

Naturally, the monopolist will pursue the largest profits in the market by offering an optimal pricing strategy. In addition, we also assume that the monopolist can grasp the information network between customers, further enabling control of the formation of the referral network. In fact, it is not that surprising that the monopolist can grasp some information about customers, especially in the era of big data, because many social media platforms and numerous online shopping websites have been continuously collecting customer information and can sell the relevant information to the monopolist.

### Customer sequence embedded in the information network

It is a common phenomenon that some customers purchase a good earlier than others and then are likely to advertise that good along their local information network. To illustrate this phenomenon, we first divide all the customers into the following two categories: the leader category and the follower category, as the basic case. Further, to simplify their description, the first *m* consumers numbered from 1 to *m* are set as the leaders and the remaining *N*−*m* consumers, numbered from *m*+1 to *N*, are set as the followers in our basic model, and their sets are denoted as *a* and *b*, respectively.

The ideal case is to divide the customers into two categories, as mentioned above. However, in real markets, customers can comprise more than two categories in their purchasing sequence. To this end, the whole *N* customers are divided into *k* parts, where *k* is an integer and *k*≥2. According to their purchasing order, the *k* parts of customers are denoted as *t*_1_, *t*_2_, ⋯, *t*_*k*_, consecutively. As a result, these parts constitute the sequence set *T*≔{*t*_1_,*t*_2_,⋯,*t*_*k*_} that satisfies *t*_*i*_∩*t*_*j*_ = Φ, ∀*i*≠*j* and |∪_1≤*i*≤*k*_*t*_*i*_| = *N*.

The two types of participants—the monopolist and the customers with the purchasing sequence—form a game in which the monopolist decides the price offered to each customer and the customers decide their consumptions. In our model, when the leaders make their consumption decision, they will consider the responses of the monopolist and the followers; when the followers make their consumption decision, the leaders’ consumption is known information for them, and they will only consider the response of the monopolist. To clarify, we first discuss the basic case and then extend it to the complicated case. Note that the solutions of these cases establish a theoretical foundation to measure the value of leading customers and, further, to propose an applicable referral mechanism in the context of network externalities, in general, and crowdfunding-based marketing patterns, in particular.

## Basic model, solutions and discussions

### Solution process for the basic model

Note that the basic model considers that all the customers are only divided into two categories, i.e., the leader set and the follower set. Hence, following the work of Candogan et al. [13], Bloch and Quérou [14] and Zhou and Chen [15], the whole solution process for the basic model consists of three steps.

#### *Step 1*: Solving the followers’ consumption strategy

Let $x^{a}≔{\left(x_{1}^{a},x_{2}^{a},\ldots ,x_{m}^{a}\right)}^{T}$ and $x^{b}≔{\left(x_{m+1}^{b},x_{m+2}^{b},\ldots ,x_{N}^{b}\right)}^{T}$ denote the leaders’ and the followers’ consumption vectors, respectively. When the followers make their consumption strategy, **x**^*a*^ is regarded as known for them by considering that the followers can directly observe the leaders’ order, which is announced on the online platform. Accordingly, the followers’ consumption strategy can be achieved by maximizing their utilities, given the prices offered by the monopolist and the leaders’ consumption, i.e.,

$$x_{j}^{b}=\underset{y_{j}^{b}\in [0,\infty )}{\arg\;\max}\;u_{j}(y_{j}^{b},x_{-j}^{b}|x^{a},p_{j}),\;(j=m+1,m+2,\ldots ,N)$$ (3)

As a result, the solution of the above formula can lead to the following optimal response function:

$$x^{b}=f_{1}(x^{a},p)$$ (4)

where the vector **p** consists of the offered price of each customer and *f*_1_ is a quasi-linear function that considers the quadratic form of the utility function. The optimal response function allows **x**^*b*^ to be explicitly expressed by the variables **x**^*a*^ and **p**, which lay the foundation for the next stages and further solutions.

#### *Step 2*: Solving the leaders’ consumption strategy

By substituting Eq (4) into the leaders’ utility function *u*_*i*_(**x**^*a*^,**x**^*b*^,**p**) (*i* = 1,2,…,*m*), the leaders’ utilities can be transferred as the function of **x**^*a*^ and **p**, i.e., *v*_*i*_(**x**^*a*^,**p**)≔*u*_*i*_(**x**^*a*^,*f*_1_(**x**^*a*^,**p**),**p**). Accordingly, the leaders’ consumption strategy can be achieved by maximizing *v*_*i*_(**x**^*a*^,**p**) with the following specific expression:

$$x_{i}^{a}=\underset{y_{i}^{a}\in [0,\infty )}{\arg\;\max}\;v_{i}\left(y_{i}^{a},x_{-i}^{a},p\right),\;(i=1,2,\ldots ,m)$$ (5)

Similar to the solution process of *Step 1*, Eq (5) also leads to the optimal response function of the leaders’ consumptions to the offered prices, i.e.,

$$x^{a}=f_{2}(p)$$ (6)

where *f*_2_ is also a quasi-linear function following the same reasons of *f*_1_. Then, by substituting Eq (6) into Eq (4), **x**^*b*^ can further be simplified as the function of the offered prices, specifically as follows:

$$x^{b}≔f_{3}(p)=f_{1}(f_{2}(p),p)$$ (7)

#### *Step 3*: The monopolist’s pricing strategy

Recalling Eq (2), the profit function of the monopolist can further be expressed as the function of the price vector **p** by substituting Eqs (6) and (7), i.e.,

$$b(p)=p^{T}\left(\begin{array}{c} x^{a}\\ x^{b} \end{array}\right)=p^{T}\left(\begin{array}{c} f_{2}(p)\\ f_{3}(p) \end{array}\right)$$ (8)

Therefore, the optimal pricing strategy of the monopolist can be achieved immediately by maximizing the profit *b*(**p**) in Eq (8), i.e.,

p*=argmaxb(p) (9)

Next, the optimal consumption strategies **x**^a^* and **x**^b^* can be achieved by substituting p* into Eqs (6) and (7), respectively. Note that the price in the optimal pricing strategy is allowed to be less than *c* or even less than 0; thus, no constraints are set for the targeted pricing strategy, because we allow the monopolist to pay some consumers if the total profit can be raised.

### Assumptions and solutions of basic model

The comprehensive network **g** defined in **Section 2.1** is divided into four parts by the two categories of customers as follows:

$$g=\left[\begin{array}{cc} g^{aa} & g^{ab}\\ g^{ba} & g^{bb} \end{array}\right]$$ (10)

Based on this expression, Assumptions 1 and 2 are discussed first to guarantee reasonable solutions.

[**Assumption 1**] Each customer’s preference parameter *β*_*i*_ is assumed to be large enough to guarantee that the two matrixes $\Lambda_{\beta }^{b}-g^{bb}$ and $\Lambda_{\beta }^{a}-g^{aa}-g^{ab}A^{b}g^{ba}-diag(g^{ab}A^{b}g^{ba})$ are invertible, where $\Lambda_{\beta }^{a}≔diag(\beta_{1},\beta_{2},\cdots ,\beta_{m})$, $\Lambda_{\beta }^{b}≔diag(\beta_{m+1},\beta_{m+2},\cdots ,\beta_{N})$, $A^{b}≔{\left[\Lambda_{\beta }^{b}-g^{bb}\right]}^{-1}$, and *diag*(**g**^*ab*^**A**^*b*^**g**^*ba*^) denotes the diagonal matrix consisting of the diagonal elements of **g**^*ab*^**A**^*b*^**g**^*ba*^.

**Assumption 1** guarantees that the law of diminishing marginal utility plays a dominant role even if the network effects are considered. Generally, this assumption is often met in the real market because the customer’s decision is often, at least to some extent, rational. Otherwise, if the influence of the network effect plays a dominant role, some customers will buy as many of the goods as possible. In most common cases, the extreme phenomenon seldom occurs in the real market, thus, **Assumption 1** guarantees a common case in real markets.

[**Assumption 2**] *α*_*i*_>*c* holds for all the customers.

**Assumption 2** guarantees that the achieved optimal consumption vector is no less than zero. In fact, this assumption means that all the concerned customers in the market truly need the good offered by the monopolist. It is true that some customers do not need the good; however, we do not consider these customers, as they do not participate in the crowdfunding project.

Based on the two assumptions and the three steps in Section 3.1, the results of the basic model are achieved below.

[**Result 1**] (**Solutions of basic model**). When the customers are divided into two categories—the leader set and follower set—the optimal consumption vector **x**_*se*_ of the customers is as follows:

$$x_{se}={\left[M^{-1}+M^{-T}\right]}^{-1}(\alpha -c1)$$ (11)

the optimal offered price vector **p**_*se*_ is as follows:

$$p_{se}=\alpha -M^{-1}{\left[M^{-1}+M^{-T}\right]}^{-1}(\alpha -c1)$$ (12)

and, accordingly, the largest profit *π*_*se*_ gained by the monopolist is as follows:

$$\pi_{se}=\frac{{\left(\alpha -c1\right)}^{T}{\left[M^{-1}+M^{-T}\right]}^{-1}(\alpha -c1)}{2}$$ (13)

Here, matrix **M** has the following specific form:

$$M=\left[\begin{array}{cc} B_{se}^{a} & B_{se}^{a}g^{ab}A^{b}\\ A^{b}g^{ba}B_{se}^{a} & A^{b}+A^{b}g^{ba}B_{se}^{a}g^{ab}A^{b} \end{array}\right]$$ (14)

***Proof***. See **S1 Appendix** for details.

It is worth noting that the achieved optimal solutions are all highlighted by the subscript “*se*” to differentiate between the results of the *simultaneous-move game*.

### Relationship with the simultaneous-move game

The basic model belongs to a *sequential-move game*; thus, an interesting question we want to explore is whether a *sequential-move game* can be transformed into an equivalent *simultaneous-move game*, where the “equivalent” means the two types of games have identical results. If the answer is yes, we can further cope with the extended model with a complicated customer sequence by considering that the equivalent *simultaneous-move game* is comparatively easy to solve.

To this end, we first recall the work of Candogan et al. [13] on the *simultaneous-move game* under a similar model setting to ours, except that all the customers in Candogan et al. [13] make their consumption decisions simultaneously. To distinguish the *sequential-move game*, we use the subscript “*si*” to denote the *simultaneous-move game*, and the relevant results of Candogan et al. [13] are listed below.

[**Result 2**] (The *simultaneous-move game* dealt with by Candogan et al. [13]). The optimal consumption vector **x**_*si*_ of all the customers is as follows:

$$x_{si}={\left[A^{-1}+A^{-T}\right]}^{-1}(\alpha -c1)$$ (15)

where **A**≔[Λ_**β**_−**g**]^−1^ and Λ_**β**_≔*diag*(*β*_1_,*β*_2_,⋯,*β*_*N*_). The optimal offered price vector **p**_*si*_ is as follows:

$$p_{si}=\alpha -A^{-1}{\left[A^{-1}+A^{-T}\right]}^{-1}(\alpha -c1)$$ (16)

and accordingly, the largest profit *π*_*si*_ gained by the monopolist is as follows:

$$\pi_{si}=\frac{{\left(\alpha -c1\right)}^{T}{\left[A^{-1}+A^{-T}\right]}^{-1}(\alpha -c1)}{2}$$ (17)

By comparing Result 1 and Result 2, we find that the two results share similar mathematical expressions, except for matrixes **M** and **A**. Accordingly, the key to linking the two types of games is to uncover the relation between the two matrixes **M** and **A**. To answer this question, matrix **A** is further re-expressed as follows:

$$A={\left[\Lambda_{\beta }-g\right]}^{-1}={\left[\begin{array}{cc} \Lambda_{\beta }^{a}-g^{aa} & -g^{ab}\\ -g^{ba} & \Lambda_{\beta }^{b}-g^{bb} \end{array}\right]}^{-1}=\left[\begin{array}{cc} B_{si}^{a} & B_{si}^{a}g^{ab}A^{b}\\ A^{b}g^{ba}B_{si}^{a} & A^{b}+A^{b}g^{ba}B_{si}^{a}g^{ab}A^{b} \end{array}\right]$$ (18)

where $B_{si}^{a}≔{\left[\Lambda_{\beta }^{a}-g^{aa}-g^{ab}A^{b}g^{ba}\right]}^{-1}$. Thus, the two matrixes **M** and **A** share a similar structure, except for the difference between $B_{se}^{a}$ and $B_{si}^{a}$. Now, by focusing on the difference between $B_{se}^{a}$ and $B_{si}^{a}$, an additional term *diag*(**g**^*ab*^**A**^*b*^**g**^*ba*^) is found in $B_{se}^{a}$. The subtle difference inspires us to transform the matrix **g** to equate the *sequential-move game* with the *simultaneous-move game* in our model setting. As a result, let **g**' be the transformed matrix from **g**, and its specific expression is provided in **Lemma 1**.

[**Lemma 1**] (The specific expression of **g**'). Let **g**^*aa*^' = **g**^*aa*^ + *diag*(**g**^*ab*^**A**^*b*^**g**^*ba*^) and the remaining three parts in **g**' remain identical to the corresponding parts in **g**, then the optimal consumption, the optimal pricing strategy and the optimal profit achieved from the simultaneous-move game under **g**' are equal to those from the sequential-move game under **g**.

***Proof***. See **S2 Appendix** for details.

As shown in **Lemma 1**, when customer *i* is a leading customer, the effect of his/her leading position in the *sequential-move game* can be reflected by adding some value to *g*_*ii*_ in the corresponding *simultaneous-move game*. Note that before the matrix transformation, *g*_*ii*_ = 0 according to our model setting. **Lemma 1** is important in understanding the relationship between the *simultaneous-move game* and the *sequential-move game* in our model setting. In addition, the matrix **M** obtained in the process of the *sequential-move game* is not convenient for mathematical expression and theoretical analysis, therefore, it is meaningful to find an equivalent *simultaneous-move game* to simplify the mathematical analysis. Lastly, the idea of matrix transformation is useful not only for the case in which the customers are divided into two categories but also for the case in which the customers are divided into multiple categories according to their purchasing sequences.

## Discussion

Unlike the results reported in Ballester et al. [12] and Zhou and Chen [15], the optimal consumption of our model is related to the matrix (**g**'+**g**'^T^)/2 rather than the matrix **g** or **g**'. The difference originates from the consideration of the monopolist and the pricing mechanism. Further, the difference will vanish when the matrix **g** or **g**' is symmetric according to their mathematical expressions. Thus, the intuition behind the above finding is that the monopolist’s power of price discrimination is rooted in the asymmetry of the influence matrix. Note that **g**'−**g**'^T^ measures the difference in influence between each pair of customers, and then, by following the interpretation provided in Candogan et al. [13], the offered price for one customer is positively affected by how much the customer is influenced by her central peers; whereas the price is meanwhile negatively affected by the influence the customer exerts on central agents. Therefore, if one customer can influence the others much more and be influenced by the others much less, the offered price for the customer will be much more favorable, which can be interpreted as the price compensation for customers who exert influence.

## Extended model and discussions

### Solutions of extended model

As we have stated, the customer sequence always contains multiple categories rather than the two categories discussed in the basic model. Accordingly, when the customer sequence contains *k* parts (*k*≥2) according to their purchasing time, we denote that these parts constitute the sequence set *T*≔{*t*_1_,*t*_2_,⋯,*t*_*k*_}. Thus, the above proven **Lemma 1** directly guarantees that the following **Result 3** holds.

[**Result 3**] (**Solutions of extended model**). Given the customers’ purchasing sequence set *T*, the optimal consumption vector **x**_*T*_, the optimal price vector **p**_*T*_ and the optimal profit *π*_*T*_ in this extend case have the following specific expressions:

$$x_{T}={\left(\Lambda_{\beta }^{}-\frac{g_{T}+g_{T}{}^{T}}{2}\right)}^{-1}\left(\frac{\alpha -c1}{2}\right)$$ (19)

$$p_{T}=\frac{\alpha +c1}{2}+\frac{\left(g_{T}-g_{T}{}^{T}\right)}{2}{\left(\Lambda_{\beta }^{}-\frac{g_{T}+g_{T}{}^{T}}{2}\right)}^{-1}\left(\frac{\alpha -c1}{2}\right)$$ (20)

and

$$\pi_{T}={\left(\frac{\alpha -c1}{2}\right)}^{T}{\left(\Lambda_{\beta }^{}-\frac{g_{T}+g_{T}{}^{T}}{2}\right)}^{-1}\left(\frac{\alpha -c1}{2}\right)$$ (21)

Here, the relationship between **g**_*T*_ and **g** is as follows:

$$g_{T}^{t_{k}t_{k}}=g^{t_{k}t_{k}}\;\mathrm{and}\;g_{T}^{t_{j}t_{j}}=g^{t_{j}t_{j}}+diag(g^{t_{j}(\cup_{j+1\leq i\leq k}t_{i})}A_{T}^{\cup_{j+1\leq i\leq k}t_{i}}g{(}^{\cup_{j+1\leq i\leq k}t_{i})t_{j}}),\;j=k-1,k-2,\cdots ,1$$ (22)

where $A_{T}^{\cup_{j+1\leq i\leq k}t_{i}}≔{\left(\Lambda_{\beta }^{}-g_{T}^{\left(\cup_{j+1\leq i\leq k}t_{i})(\cup_{j+1\leq i\leq k}t_{i}\right)}\right)}^{-1}$.

***Proof***. See **S3 Appendix** for details.

**Result 3** provides the recursive form of receiving the targeted matrix **g**_*T*_, which extends the basic model. Thus far, no matter how many categories are contained by the customer sequence, the proven **Result 3** is the approach for transforming it into the equivalent *simultaneous-move game* to provide direct results.

### Comparisons with the simultaneous-move game

In our model setting, if all the customers make their consumption decisions simultaneously, how do each customer’s optimal consumption and the monopolist’s optimal profit change? The following **Lemma 2** provides the answer, where two matrixes **x** and **y** satisfy **x**≥**y** if and only if **x**(*i*,*j*)≥**y**(*i*,*j*), ∀*i*,*j*∈*N*.

[**Lemma 2**] (**Comparing the customers’ optimal consumption vectors and the monopolist’s optimal profits of two games**). Given the customers’ purchasing sequence set *T*, let **x**_*se*_ (or **x**_*si*_) denote the optimal consumption vector and *π*_*se*_ (or *π*_*si*_) denote the highest profit of the monopolist in the *sequential-move game* (or *simultaneous-move game*). Thus, it holds that **x**_*se*_≥**x**_*si*_ and *π*_*se*_≥*π*_*si*_. In addition, when **g** is an upper or a lower triangular matrix, it holds that **x**_*se*_ = **x**_*si*_ and *π*_*se*_ = *π*_*si*_.

***Proof***. See **S4 Appendix** for details.

Furthermore, from the perspective of the monopolist, the existence of leading customers will increase, or at least not decrease, the monopolist’s profit; thus, cultivating some leading customers will benefit the monopolist. Additionally, Lemma 2 lays a foundation to measure the value of leading customers, which is the focus of the next section.

Before discussing this value, however, we further pay attention to the offered price vectors **p**_*se*_ and **p**_*si*_ in the two types of games. By recalling Eqs (16) and (20), the fact that **g**−**g**^T^ = **g**_*T*_−**g**_*T*_^T^ guarantees the following:

$$p_{se}-p_{si}=\underset{\begin{array}{c} \mathrm{Effect}\;\mathrm{from}\\ \mathrm{Matrix}\;\mathrm{asymmetry} \end{array}}{\underset{︸}{\frac{\left(g-g^{T}\right)}{2}}}\underset{\mathrm{Effect}\;\mathrm{from}\;\mathrm{adding}\;\mathrm{leading}\;\mathrm{consumers}}{\underset{︸}{\left({\left(\Lambda_{\beta }^{}-\frac{g_{T}+g_{T}{}^{T}}{2}\right)}^{-1}-{\left(\Lambda_{\beta }^{}-\frac{g+g^{T}}{2}\right)}^{-1}\right)}}\underset{\begin{array}{c} \mathrm{Effect}\;\mathrm{from}\\ \mathrm{inner}\;\mathrm{preference} \end{array}}{\underset{︸}{\left(\frac{\alpha -c1}{2}\right)}}$$ (23)

which shows that the difference between **p**_*se*_ and **p**_*si*_ originates from the following three effects: matrix asymmetry, the addition of leading consumers and inner preference. Accordingly, the properties of the relation between **p**_*se*_ and **p**_*si*_ are obtained in **Lemma 3**.

[**Lemma 3**]. (**Comparing the offered price vector of two games**). (1) When **g** is a symmetric matrix, **p**_*se*_ = **p**_*si*_; (2) when **g** is an upper or a lower triangular matrix, **p**_*se*_ = **p**_*si*_.

***Proof***. The first part is obvious by recalling Eq (22), and the second part originates from the proof process of Lemma 2, where it is shown that **g**_*T*_−**g** if **g** is an upper or a lower triangular matrix.

Moreover, the existence of leading customers **does not** guarantee that **p**_*se*_≥**p**_*si*_ or **p**_*se*_<**p**_*si*_. However, in most cases, adding or cultivating the leading customers will increase the monopolist’s profit, which can be paid special attention to by introducing the referral mechanism.

### Discussion

From **Lemma 1** and **Result 3**, we find that only the diagonal elements of the leading customers increase compared to the original matrix **g**. Accordingly, we can also understand the effect of leading customers from the perspective of *β*_*i*_ by noting that (Λ_**β**_−(**g**'+**g**'^T^)/2)^−1^ or (Λ_**β**_−(**g**_*T*_+**g**_*T*_^T^)/2)^−1^ always appear in the result as a whole. In other words, a leading customer *i* with his/her preference parameter *β*_*i*_ in the *sequential-move game* is equivalent to a customer with a new preference parameter *β*_*i*_'≤*β*_*i*_ in the *simultaneous-move game*. Namely, the advantage of a leading position in the *sequential-move game* can be transferred to be a decrease in the preference parameter *β*_*i*_ in the simultaneous-move game. Recalling the adopted utility function in Eq (1), *β*_*i*_ measures the decreasing effect of marginal utility so that a smaller value means a lower decreasing effect.

## The value of leading customers

This section aims to utilize the above proven lemmas and results to provide an applicable network-based referral mechanism. Because **Lemma 2** has demonstrated that adding or cultivating some leading customers will increase the monopolist’s profit in most cases, we can accordingly define the value of leading customers from the perspective of increased profit. Next, recalling the designed referral network in Eq (1), we can use the defined value of leading customers to reward the leading customers to encourage them to form the referral network. Based on this basic idea, we further explore the properties and present the potential application of the provided network-based referral mechanism.

### Definition and example

The monotonicity of the monopolist’s profit helps us measure the value of the leading customers, and then, **Definition 1** provides the formal expressions.

[**Definition 1**] Given the customer sequence *T*≔{*t*_1_,*t*_2_,⋯,*t*_*j*_,*t*_*j*+1_,⋯,*t*_*k*_}, we denote *T*(*j*)≔{*t*_1_,*t*_2_,⋯,*t*_*j*_, (*t*_*j*+1_∪*t*_*j*+2_∪⋯∪*t*_*k*_)} and define *T*(0)≔*T*; then the value of leading customer set *t*_*j*_ is denoted as *value*(*t*_*j*_) with the following expression:

$$value(t_{j})≔\pi_{se}(g_{T(j)})-\pi_{se}(g_{T(j-1)})$$ (24)

where the profit function *π*_*se*_(⋅) has been shown in Eq (21).

Next, two basic network structures are presented as examples to provide an intuitive understanding of the definition and to further illustrate some interesting findings.

**Fig 1:**
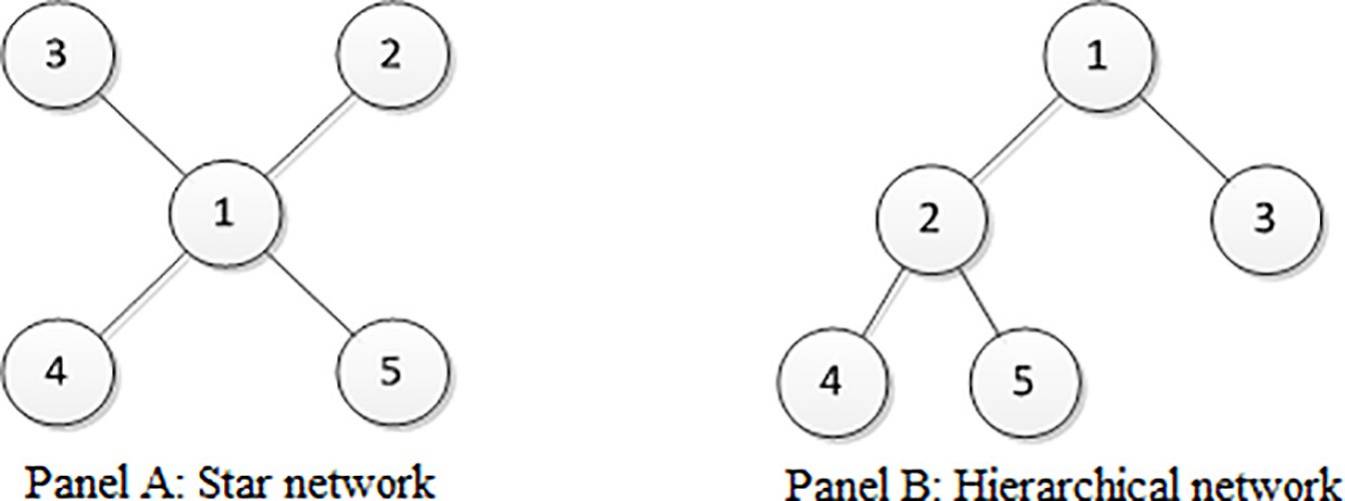
Two basic network structures.

As shown in Fig 1, the adopted two basic networks contain the same number of nodes and links, which can be regarded as two typical basic structures of a complex network. According to **Definition 1**, Table 1 displays the values of the leading customer sets under different customer sequences.

**Table 1:** The values of the leading customer sets under different customer sequences.

Case No.	customer sequence	star network		hierarchical network	
		value of leading customer	profit	value of leading customer	profit
1	{1,2,3,4,5}	—-	0.3929	—-	0.3800
2	{1},{2,3,4,5}	value({1}) = 0.0453	0.4382	value({1}) = 0.0124	0.3924
3	{2},{1,3,4,5}	value({2}) = 0.0048	0.3977	value({2}) = 0.0249	0.4049
4	{2},{1,3},{4,5}	value({2}) = 0.0048; value({1,3}) = 0.0212	0.4189	value({2}) = 0.0249; value({1,3}) = 0	0.4049
5	{2},{4,5},{1,3}	value({2}) = 0.0048; value({4,5}) = 0.0090	0.4067	value({2}) = 0.0249; value({4,5}) = 0	0.4049
6	{1,2},{3,4,5}	value({1,2}) = 0.0321	0.4250	value({1,2}) = 0.0214	0.4014

In this example, *α*_*i*_ = 1, *β*_*i*_ = 5 and *c*_*i*_ = 0, for any *i*∈*N*.

By comparing these results, the following findings can be summarized. Although two networks share the same number of nodes and links, the profit of the monopolist is different according to case No. 1. Accordingly, the network structure in itself can influence profits, and thus, some network structures are preferred by the monopolist. In addition, by comparing case No. 2 and No. 3, choosing different leading customers will lead to different profits; thus, the strategy of choosing the leading customer set is meaningful for the monopolist in increasing the achieved profit. Moreover, the difference between case No. 4 and No. 5 illustrates that the order of the customer sequence will be a factor that influences the profit of the monopolist. Accordingly, when the customer sequence comprises more than two sets, it will be an interesting problem to determine the optimal customer sequence to achieve the largest profit. Finally, by comparing cases No. 6 and No. 2, if more nodes are chosen as the leading customers, then the profit must not increase. Specifically, compared with case No. 2, the profit decreases when node 2 is added into the leading customer set in the star network, whereas the hierarchical network shows the opposite. Accordingly, the relationship between the achieved profit and the amount of leading customers is not monotonous.

### Network-based referral strategy

Aside from the above interesting findings, this subsection focuses on coping with exactly how many rewards should be allocated to the leading customers if they benefit the monopoly by increasing the purchase of the targeted product, especially in the crowdfunding-based marketing pattern. Note that the approach of allocating rewards along the referral network is the core of the referral mechanism. Specifically, as an advertising strategy, the monopolist rewards the existing customers who successfully recommend some newcomers to participate in the crowdfunding campaign. One direct problem is exactly how much of a reward should be allocated. As we have explained, the defined value of leading customers can be a tool to cope with the problem. On the one hand, when a newcomer participates in the crowdfunding campaign, the values of the leading customers are likely to change, which can quantitatively reflect the effect of a successful recommendation. On the other hand, the change in the values of the leading customers originates from the change in the monopolist’s profits, so it is helpful for the design of the marketing mechanism, especially when social learning becomes increasingly important with the development of modern crowdfunding.

Before going into detail, we first answer the following fundamental question: will adding a new customer increase or at least not decrease all the leading customers’ values regardless of their sequence positions? If the answer is YES, we can argue that each leading customer with a direct or indirect influence on the newcomer will benefit from the arrival of a new customer. **Lemma 4** provides the answer.

[**Lemma 4**] Given the customer sequence *T*≔{*t*_1_,*t*_2_,⋯,*t*_*j*_,*t*_*j*+1_,⋯,*t*_*k*_} and the comprehensive network **g** shown in Eq (1B), a newcomer denoted as *t*_*k*+1_ is added into the customer sequence; then, the new customer sequence is denoted as *T*_+1_≔{*t*_1_,*t*_2_,⋯,*t*_*j*_,*t*_*j*+1_,⋯,*t*_*k*_,*t*_*k*+1_}. As a result, *value*(*t*_*j*_|*T*_+1_)≥ *value*(*t*_*j*_|*T*) holds for any *j*∈{1,2,⋯,*k*}, where *value*(*t*_*i*_|*T*_+1_) and *value*(*t*_*i*_|*T*) represent the value of leading customer set *t*_*j*_ in the customer sequences *T*_+1_ and *T*, respectively.

***Proof***. See **S5 Appendix** for details.

**Fig 2:**
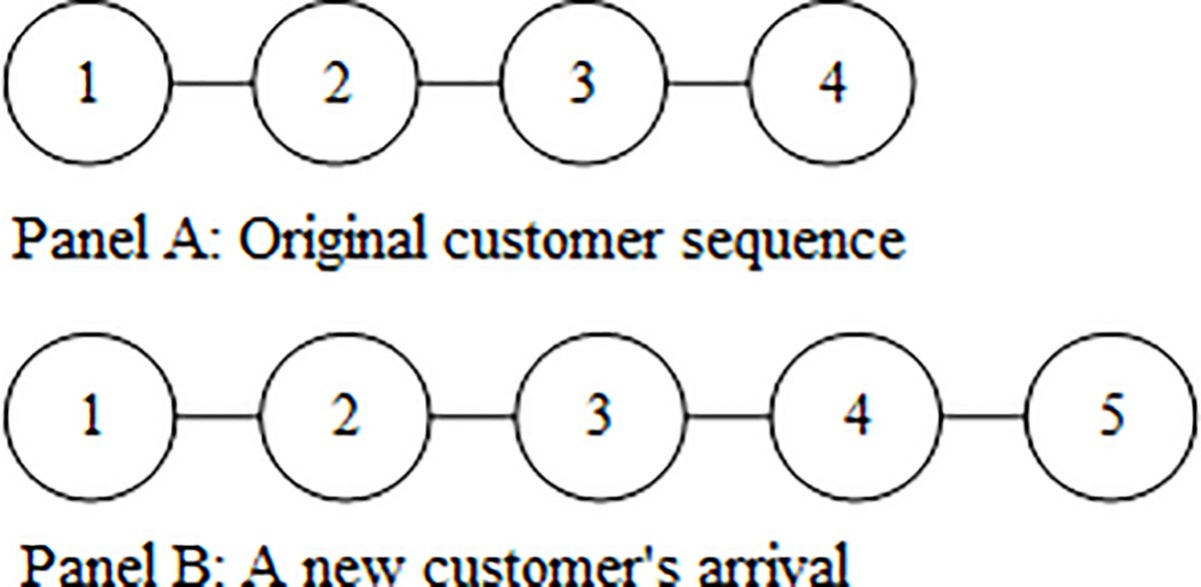
Two customer sequences.

**Lemma 4** actually points out that when a newcomer is added at the end of the customer sequence, all the leading customers of the newcomer will not decrease their leading values. Based on the theoretic basis of **Lemma 4**, Eq (24) further guides us to measure the newcomer’s contributions to increasing each leading customer’s value. To clarify, one example is provided below, where Panel A of Fig 2 displays a simple comprehensive network comprising four customers with their customer sequence {{1},{2},{3},{4}}, and Panel B of Fig 2 displays a new customer’s arrival and the change of their customer sequence to {{1},{2},{3},{4},{5}}. Next, given that all the customers share identical preference parameters, each leading customer’s value and their changes upon the addition of a newcomer are listed in Table 2.

**Table 2:** Each leading customer’s value and the change.

Panel A	Leading value	Panel B	Leading value	Increased value
{1}	0.0038	{1}	0.0038	+0.0000
{2}	0.0057	{2}	0.0058	+0.0001
{3}	0.0056	{3}	0.0063	+0.0007
{4}	0	{4}	0.0057	+0.0057
—-	—-	{5}	0	—-

*α*_*i*_ = 1, *β*_*i*_ = 5, *c*_*i*_ = 0 and *η* = 1 for any *i*∈*N*.

Table 2 validates the result of **Lemma 4**, where all the leading customer’s values increase. As displayed in Fig 2, new customer No. 5 is recommended by customer No. 4, so that the pair have a direct influence. Furthermore, the leading customers at different sequence positions truly increase at different values; the value directly influenced by the newcomer has the largest increase and is called **the rule of “closeness”**, and an earlier leading customer has a lower value increase, which is called **the rule of “decreasing”**. By recalling the background provided in the Introduction, another finding emerging from this example is that not only does the one who directly recommends the newcomer benefit from the newcomer’s arrival, but the earlier leading customers also benefit, although they only have indirect influences.

Inspired by this simple example, we pay further close attention to the general rule of the proposed network-based referral strategy. Thus far, we know that adding a newcomer will increase the leading values of these customers along the referral line and we also present the approach of calculating the increased leading values. Next, we illustrate the two rules discovered in the above simple example to facilitate the crowdfunding initiator to reward the related leading customers for their successful recommendations. Furthermore, our model only allows one newcomer to accept one existing customer’s recommendation, although the newcomer can actually receive information from several customers; in other words, each customer has at most one referrer. The abovementioned mechanism of acceptance will ease the allocations of rewards because all the related leading customers constitute a line network. Deepening the analysis of the above simple example, **Property 1** provides the general rules for allocating the increased profits from the newcomer in our proposed network-based referral strategy.

[**Property 1**] (**General rules of the proposed network-based referral strategy**). The rule of “closeness” and the rule of “decreasing” should be adopted when allocating the increased profits from the newcomer along the line network consisting of the related leading customers, given that all the customers share identical preference parameters.

***Proof***. See **S6 Appendix** for details.

In summary, customers can be encouraged by the rewards obtained when they successfully recommend newcomers to participate. Especially with the development of the sharing economy, for example, as in a crowdfunding-based marketing model, it becomes more convenient and even important for customers to utilize their social networks to recommend the targeted product. This subsection provides an alternative approach for encouraging customers to make successful recommendations, as well as determining exactly how much profit should be allocated. More precisely, the two rules demonstrated in **Property 1** can qualitatively guide the monopolist in how to allocate the additional profits to each leading customer when a new customer enters the market. Specifically, the customer who directly recommends and affects the newcomer will be allocated most of the profits because this customer contributes most to the new customer’s entrance into the market, and the earlier customer in the line network should be allocated much less, given the longer distance to the newcomer. In addition, the proven **Lemma 2** provides a quantitative approach to coping with how much profit should actually be returned based on the calculated values of the leading customers.

### Discussion

Recalling the utility function defined in Eq (1A), there are two effects discussed and considered. One is the influence of the referrers and the other is the influence of the successfully recommended followers. In a real market, modern technology allows us to record the information of a successful recommendation; thus, the referral network ***R*** is clear, especially in the online crowdfunding-based marketing platform. The remaining problem is how the strength parameter *η* affects the strategy of allocation, and Property 2 provides the answer.

[**Property 2**]. (**The effect of strength parameter** *η*). When *η* = 0, each leading customer’s value equals 0 in our model setting. In addition, each leading customer’s value increases with the rise of *η*.

***Proof*.** When *η* = 0, the comprehensive matrix **g** becomes a lower triangular matrix, and Lemma 2 guarantees that each leading customer’s value equals 0 in our model setting. In addition, Eqs (21) and (22) guarantee that a larger *η* means larger diagonal elements in **g**_*T*_, which, as a result, will cause the value of each leading customer to increase.

**Property 2** points out that if the leading customers are not affected by their followers’ consumption decisions (i.e., *η* = 0), the leading values vanish, i.e., the leading positions do not take effect. However, if the leading customers are greatly influenced by their followers’ consumption decisions (i.e., a larger *η*), the leading positions become much more valuable. In a real market, when some customers accept the recommendation, the referrer truly increases his/her utility because some peers agree with the referrer regarding the targeted project; meanwhile, the much larger amounts consumed by the followers means a higher increase in utility. Thus, *η* should be larger than 0 in a real market in most cases. Following the result of **Property 2**, if we can obtain each customer’s information about his/her reaction to their peers’ consumption decisions in advance, the ones with a much greater reaction should be chosen as the leading ones from the perspective of *η* when the other influence factors are identical.

## Conclusions

Currently, crowdfunding is gradually becoming a modern marketing mode via the Internet. Therefore, we aim to provide feasible marketing strategies to promote modern crowdfunding-based crowdfunding models. To this end, we first analyzed the features of modern crowdfunding-based marketing models and find the following three features: monopoly structure, network externalities and customer sequence. Building on the work of Candogan et al. [13], Zhou and Chen [15] and Zhou and Chen [18], we defined the values of leading customers by solving the game models (i.e., basic model and extended model) contained within the above three features. Based on the solution of a basic model comprising only of leaders and followers, the relationship between the *simultaneous-move game* and *sequence-move game* is revealed; then, the extended model, whose customer sequence consists of more than two sets, is solved. According to the defined leading customer’s values, we further focus on providing a network-based referral mechanism in the crowdfunding-based market model by exploring how to allocate rewards along the customer sequence according to their leading values and by uncovering the rules and properties of the proposed network-based referral mechanism.

The main marketing strategies for promoting crowdfunding-based marketing patterns are summarized as follows. When a customer sequence with more than two customer sets is formed, the crowdfunding initiator can also earn greater profits by rewarding the leading customers for utilizing their social networks for providing recommendations. The demonstrated strategies for allocating the rewards have the following rules: (1) the customer who directly recommends the newcomer will be allocated the most additional profits and (2) the earlier leading customer in the line network should be allocated fewer profits, or exactly exponentially decreasing profits. We tend to believe that the proposed quantitative strategies are easy to operate in a real market.

## Supporting information

S1 Appendix (DOCX)

S2 Appendix (DOCX)

S3 Appendix (DOCX)

S4 Appendix (DOCX)

S5 Appendix (DOCX)

S6 Appendix (DOCX)
