# Supplementary material for: The value of leading customers in a crowdfunding-based marketing pattern
Source: PLoS One. 2019 Apr 15;14(4):e0215323. doi: 10.1371/journal.pone.0215323 (PMC6464345; doi:10.1371/journal.pone.0215323)
Supplement: S1 Appendix — (DOCX) [file pone.0215323.s001.docx]

Proof of Result 1.

By following the three solution steps mentioned in Section 3.1, the result of ***Step 1***, namely, the specific expression of Equation (4), is $\mathbf{x}^{b}=[\Lambda_{\mathbf{\beta}}^{b}-\mathbf{g}^{bb}]^{-1}[\mathbf{\alpha}^{b}-\mathbf{p}^{b}+\mathbf{g}^{ba}\mathbf{x}^{a}]$. Similarly, the result of ***Step 2*** is $\mathbf{x}^{a}=\left[\Lambda_{\mathbf{\beta}}^{a}-\mathbf{g}^{aa}-\mathbf{g}^{ab}\mathbf{A}^{b}\mathbf{g}^{ba}-diag(\mathbf{g}^{ab}\mathbf{A}^{b}\mathbf{g}^{ba})\right]^{-1}\left(\mathbf{\alpha}^{a}-\mathbf{p}^{a}+\mathbf{g}^{ab}\mathbf{A}^{b}(\mathbf{\alpha}^{b}-\mathbf{p}^{b})\right)$.

Next, substituting $\mathbf{x}^{a}$ into $\mathbf{x}^{b}$, we obtain $\mathbf{x}^{b}=(\mathbf{A}^{b}\mathbf{g}^{ba}\mathbf{B}_{se}^{a})(\mathbf{\alpha}^{a}-\mathbf{p}^{a})+(\mathbf{A}^{b}+\mathbf{A}^{b}\mathbf{g}^{ba}\mathbf{B}_{se}^{a}\mathbf{g}^{ab}\mathbf{A}^{b})(\mathbf{\alpha}^{b}-\mathbf{p}^{b})$, where $\mathbf{B}_{se}^{a}:=\left[\Lambda_{\mathbf{\beta}}^{a}-\mathbf{g}^{aa}-\mathbf{g}^{ab}\mathbf{A}^{b}\mathbf{g}^{ba}-diag(\mathbf{g}^{ab}\mathbf{A}^{b}\mathbf{g}^{ba})\right]^{-1}$. Thus far, the optimal consumptions $\mathbf{x}^{a}$ and $\mathbf{x}^{b}$ of two categories are both expressed as the function of the offered price vector $\mathbf{p}$, and then we further express them in matrix form as follows:

$\left(\begin{matrix} \mathbf{x}^{a} \\ \mathbf{x}^{b} \end{matrix}\right)=\mathbf{M}\left(\begin{matrix} \mathbf{\alpha}^{a}-\mathbf{p}^{a} \\ \mathbf{\alpha}^{b}-\mathbf{p}^{b} \end{matrix}\right)$,

where the matrix $\mathbf{M}$ takes the following form:

$\mathbf{M}=\left[\begin{matrix} \mathbf{B}_{se}^{a} & \mathbf{B}_{se}^{a}\mathbf{g}^{ab}\mathbf{A}^{b} \\ \mathbf{A}^{b}\mathbf{g}^{ba}\mathbf{B}_{se}^{a} & \mathbf{A}^{b}+\mathbf{A}^{b}\mathbf{g}^{ba}\mathbf{B}_{se}^{a}\mathbf{g}^{ab}\mathbf{A}^{b} \end{matrix}\right]$.

Finally, ***Step 3*** is conducted to achieve the solutions of the *basic model*. Specifically, the optimal consumption of the customers is as follows:

$\mathbf{x}_{se}=[\mathbf{M}^{-1}+\mathbf{M}^{-\text{T}}]^{-1}(\mathbf{\alpha}-c\mathbf{1})$,

the optimal offered pricing vector is as follows:

$\mathbf{p}_{se}=\mathbf{\alpha}-\mathbf{M}^{-1}[\mathbf{M}^{-1}+\mathbf{M}^{-\text{T}}]^{-1}(\mathbf{\alpha}-c\mathbf{1})$,

and accordingly, the largest profit gained by the monopolist is as follows:

$\pi_{se}=\frac{(\mathbf{\alpha}-c\mathbf{1})^{\text{T}}[\mathbf{M}^{-1}+\mathbf{M}^{-\text{T}}]^{-1}(\mathbf{\alpha}-c\mathbf{1})}{2}$.
