# Supplementary material for: The value of leading customers in a crowdfunding-based marketing pattern
Source: PLoS One. 2019 Apr 15;14(4):e0215323. doi: 10.1371/journal.pone.0215323 (PMC6464345; doi:10.1371/journal.pone.0215323)
Supplement: S2 Appendix — (DOCX) [file pone.0215323.s002.docx]

Proof of Lemma 1.

Replacing $\mathbf{g}$ by $\mathbf{g}'$, the consumer’s utility function in the *simultaneous-move game* is as follows:

$u_{i}(x_{i};\mathbf{x}_{-i},p_{i})=\alpha_{i}x_{i}-\frac{1}{2}\beta_{i}x_{i}^{2}+\sum_{j=1}^{N} g_{ij}'x_{i}x_{j}-p_{i}x_{i}$.

According to **Result 2** shown in Section 3.3, we immediately obtain the expressions of the optimal consumption vector and the optimal price vector by replacing $\mathbf{g}$ with $\mathbf{g}'$, respectively. Thus, $\mathbf{B}_{si}^{a}$ is further expressed as $\mathbf{B}_{si}^{a}(\mathbf{g}')$ with the following formula:

,

because the matrix transformation does not change $\mathbf{g}^{ab}$, $\mathbf{g}^{ba}$, $\mathbf{g}^{bb}$ as well as the corresponding $\mathbf{A}^{b}$. Moreover, according to the condition that =$\mathbf{g}^{aa}+$ $diag(\mathbf{g}^{ab}\mathbf{A}^{b}\mathbf{g}^{ba})$ and the expression of $\mathbf{B}_{se}^{a}$, it immediately holds that

$\mathbf{B}_{si}^{a}(\mathbf{g}')=\mathbf{B}_{se}^{a}(\mathbf{g})$,

where $\mathbf{B}_{se}^{a}$ is highlighted as the function of the matrix $\mathbf{g}$. Next, the above Equation guarantees that

$\mathbf{M}(\mathbf{g})=\mathbf{A}(\mathbf{g}')$,

where the matrix $\mathbf{M}$ and $\mathbf{A}$ are highlighted as the functions of $\mathbf{g}$ and $\mathbf{g}'$, respectively. Thus, by recalling the expressions of $\mathbf{x}_{se}$, $\mathbf{p}_{se}$, $\mathbf{x}_{si}$, $\mathbf{p}_{si}$, $\pi_{si}$ and $\pi_{se}$, **Lemma 1** holds.
