# Supplementary material for: The value of leading customers in a crowdfunding-based marketing pattern
Source: PLoS One. 2019 Apr 15;14(4):e0215323. doi: 10.1371/journal.pone.0215323 (PMC6464345; doi:10.1371/journal.pone.0215323)
Supplement: S3 Appendix — (DOCX) [file pone.0215323.s003.docx]

Proof of Result 3.

The part $t_{k}$ has no followers, so their decisions are not affected by the followers. As a result, the transformed matrix of $t_{k}$ remains unchanged; specifically, $\mathbf{g}_{T}^{t_{k}t_{k}}=\mathbf{g}^{t_{k}t_{k}}$. Then, the part $t_{k-1}$ has the follower part $t_{k}$ , therefore **Lemma 1** guarantees the following:

$\mathbf{g}_{T}^{t_{k-1}t_{k-1}}=\mathbf{g}^{t_{k-1}t_{k-1}}+diag(\mathbf{g}^{t_{k-1}t_{k}}\mathbf{A}_{T}^{t_{k}}\mathbf{g}^{t_{k}t_{k-1}})$,

which accords with **Result 1** when only two categories exist.

Next, when the part $t_{k-2}$ is considered, it has two parts of followers, i.e., $t_{k}$ and $t_{k-1}$, which have an influence on $t_{k}$. Then, the combined two parts’ new influence matrix $\mathbf{g}_{T}^{(t_{k-1}\cup t_{k})(t_{k-1}\cup t_{k})}$ can be reached as follows:

$\mathbf{g}_{T}^{(t_{k-1}\cup t_{k})(t_{k-1}\cup t_{k})}=\left[\begin{matrix} \mathbf{g}_{T}^{t_{k-1}t_{k-1}} & \mathbf{g}_{T}^{t_{k-1}t_{k}} \\ \mathbf{g}_{T}^{t_{k}t_{k-1}} & \mathbf{g}_{T}^{t_{k}t_{k}} \end{matrix}\right]=\left[\begin{matrix} \mathbf{g}_{T}^{t_{k-1}t_{k-1}} & \mathbf{g}^{t_{k-1}t_{k}} \\ \mathbf{g}^{t_{k}t_{k-1}} & \mathbf{g}^{t_{k}t_{k}} \end{matrix}\right]$,

where only the part $\mathbf{g}_{T}^{t_{k-1}t_{k-1}}$ is different from the original $\mathbf{g}^{(t_{k-1}\cup t_{k})(t_{k-1}\cup t_{k})}$. Accordingly, once the two parts are considered together, **Lemma 1** is also useful for achieving the transformed $\mathbf{g}^{t_{k-2}t_{k-2}}$, as follows:

$\mathbf{g}_{T}^{t_{k-2}t_{k-2}}=\mathbf{g}^{t_{k-2}t_{k-2}}+diag(\mathbf{g}^{t_{k-2}(t_{k-1}\cup t_{k})}\mathbf{A}_{T}^{t_{k-1}\cup t_{k}}\mathbf{g}^{(t_{k-1}\cup t_{k})t_{k-2}})$.

Repeating the above process, we can achieve the transformed $\mathbf{g}^{t_{j}t_{j}}$ ($1\leq j\leq k-3$) by considering the followers $t_{j+1}$, $t_{j+2}$, , $t_{k}$ as one whole part. Note that the whole part’s influence matrix has been transformed as $\mathbf{g}_{T}^{(\cup_{j+1\leq i\leq k}t_{i})(\cup_{j+1\leq i\leq k}t_{i})}$, where $\mathbf{g}_{T}^{t_{i}t_{i}}(j+1\leq i\leq k)$ has been achieved in the above processes. As a result, **Lemma 1** guarantees that $\mathbf{g}_{T}^{t_{j}t_{j}}=\mathbf{g}^{t_{j}t_{j}}+diag(\mathbf{g}^{t_{j}(\cup_{j+1\leq i\leq k}t_{i})}\mathbf{A}_{T}^{\cup_{j+1\leq i\leq k}t_{i}}\mathbf{g}^{(\cup_{j+1\leq i\leq k}t_{i})t_{j}})$. In all, **Result 3** holds.
