# Supplementary material for: The value of leading customers in a crowdfunding-based marketing pattern
Source: PLoS One. 2019 Apr 15;14(4):e0215323. doi: 10.1371/journal.pone.0215323 (PMC6464345; doi:10.1371/journal.pone.0215323)
Supplement: S4 Appendix — (DOCX) [file pone.0215323.s004.docx]

Proof of Lemma 2.

We first prove this lemma when all the customers are only divided into the two categories of leaders and followers. In this case, it first holds that

$\left(\Lambda_{\mathbf{\beta}}-\frac{\mathbf{g}'+\mathbf{g}'^{\text{T}}}{2}\right)^{-1}-\left(\Lambda_{\mathbf{\beta}}-\frac{\mathbf{g}+\mathbf{g}^{\text{T}}}{2}\right)^{-1}=\left(\Lambda_{\mathbf{\beta}}-\frac{\mathbf{g}+\mathbf{g}^{\text{T}}}{2}\right)^{-1}\left(\frac{\mathbf{g}'+\mathbf{g}'^{\text{T}}}{2}-\frac{\mathbf{g}+\mathbf{g}^{\text{T}}}{2}\right)\left(\Lambda_{\mathbf{\beta}}-\frac{\mathbf{g}'+\mathbf{g}'^{\text{T}}}{2}\right)^{-1}$,

and then, according to the relationship between $\mathbf{g}$ and $\mathbf{g}'$, we have

$\left(\frac{\mathbf{g}'+\mathbf{g}'^{\text{T}}}{2}-\frac{\mathbf{g}+\mathbf{g}^{\text{T}}}{2}\right)=diag\left(\begin{matrix} \mathbf{g}^{ab}\mathbf{A}^{b}\mathbf{g}^{ba} & \\ & \mathbf{0}_{(N-m)\times(N-m)} \end{matrix}\right)$,

where $\mathbf{0}_{(N-m)\times(N-m)}$ is an $(N-m)\times(N-m)$ matrix. Next, by recalling Assumption 1, all the elements are not less than 0 in the following three matrixes:

$\left(\Lambda_{\mathbf{\beta}}-\frac{\mathbf{g}+\mathbf{g}^{\text{T}}}{2}\right)^{-1}$, $diag\left(\begin{matrix} \mathbf{g}^{ab}\mathbf{A}^{b}\mathbf{g}^{ba} & \\ & \mathbf{0}_{(N-m)\times(N-m)} \end{matrix}\right)$, and $\left(\Lambda_{\mathbf{\beta}}-\frac{\mathbf{g}'+\mathbf{g}'^{\text{T}}}{2}\right)^{-1}$.

As a result, it immediately holds that

$\left(\Lambda_{\mathbf{\beta}}-\frac{\mathbf{g}'+\mathbf{g}'^{\text{T}}}{2}\right)^{-1}\geq\left(\Lambda_{\mathbf{\beta}}-\frac{\mathbf{g}+\mathbf{g}^{\text{T}}}{2}\right)^{-1}$.

Thus, note that no matter how many categories are contained in the customer sequence, the above result holds for any two divisions contained in the submatrixes. In all, **Lemma 2** holds.
