# Supplementary material for: The value of leading customers in a crowdfunding-based marketing pattern
Source: PLoS One. 2019 Apr 15;14(4):e0215323. doi: 10.1371/journal.pone.0215323 (PMC6464345; doi:10.1371/journal.pone.0215323)
Supplement: S5 Appendix — (DOCX) [file pone.0215323.s005.docx]

Proof of Lemma 4.

According to **Definition 1**, we immediately achieve the following equation:

$$value(t_{j}|T_{+1})=\left(\frac{\mathbf{\alpha}-c\mathbf{1}}{2}\right)^{\text{T}}\left(\left(\Lambda_{\mathbf{\beta}}-\frac{\mathbf{g}_{T_{+1}(j)}+\mathbf{g}_{T_{+1}(j)}^{\text{T}}}{2}\right)^{-1}-\left(\Lambda_{\mathbf{\beta}}-\frac{\mathbf{g}_{T_{+1}(j-1)}+\mathbf{g}_{T_{+1}(j-1)}^{\text{T}}}{2}\right)^{-1}\right)\left(\frac{\mathbf{\alpha}-c\mathbf{1}}{2}\right)$$

$$=\left(\frac{\mathbf{\alpha}-c\mathbf{1}}{2}\right)^{\text{T}}\left(\Lambda_{\mathbf{\beta}}-\frac{\mathbf{g}_{T_{+1}(j-1)}+\mathbf{g}_{T_{+1}(j-1)}^{\text{T}}}{2}\right)^{-1}\left(\frac{\mathbf{g}_{T_{+1}(j)}+\mathbf{g}_{T_{+1}(j)}^{\text{T}}}{2}-\frac{\mathbf{g}_{T_{+1}(j-1)}+\mathbf{g}_{T_{+1}(j-1)}^{\text{T}}}{2}\right)\left(\Lambda_{\mathbf{\beta}}-\frac{\mathbf{g}_{T_{+1}(j)}+\mathbf{g}_{T_{+1}(j)}^{\text{T}}}{2}\right)^{-1}\left(\frac{\mathbf{\alpha}-c\mathbf{1}}{2}\right),$$

Next, we add an isolated node into the given customer sequence $T$; therefore, we also obtain a new customer sequence denoted as $T_{+1}'$ with *k*+1 elements. Note that the added node is isolated so that the new formed influence matrix $\mathbf{g}'$ has the following expressions: $g_{ij}'=g_{ij}$, $g_{i(n+1)}'=0$ and $g_{(n+1)j}'=0$ for any $i,j\in\{1,2,\cdots,n\}$, and $g_{(n+1)(n+1)}'=0$.

Thus, recalling **Result 3** proven above, we immediately achieve the following two results:

(i) All the elements in $\mathbf{g}_{T_{+1}(j)}$ are no less than $\mathbf{g}_{T_{+1}(j)}'$ for any $j\in\{1,2,\cdots,k\}$, which is denoted as $\mathbf{g}_{T_{+1}(j)}\geq\mathbf{g}_{T_{+1}(j)}'$, and therefore it holds that

$\left(\Lambda_{\mathbf{\beta}}-\frac{\mathbf{g}_{T_{+1}(j)}+\mathbf{g}_{T_{+1}(j)}^{\text{T}}}{2}\right)^{-1}\geq\left(\Lambda_{\mathbf{\beta}}-\frac{\mathbf{g}_{T_{+1}(j)}'+\mathbf{g}_{T_{+1}(j)}'^{\text{T}}}{2}\right)^{-1}$;

(ii) The series of equations provided in Equation (22) guarantee that

$\frac{\mathbf{g}_{T_{+1}(j)}+\mathbf{g}_{T_{+1}(j)}^{\text{T}}}{2}-\frac{\mathbf{g}_{T_{+1}(j-1)}+\mathbf{g}_{T_{+1}(j-1)}^{\text{T}}}{2}\geq\frac{\mathbf{g}_{T_{+1}(j)}'+\mathbf{g}_{T_{+1}(j)}'^{\text{T}}}{2}-\frac{\mathbf{g}_{T_{+1}(j-1)}'+\mathbf{g}_{T_{+1}(j-1)}'^{\text{T}}}{2}\geq\mathbf{0}_{(n+1)\times(n+1)}$.

By considering the above results together, we further obtain $value(t_{j}|T_{+1})\geq value(t_{j}|T_{+1}')$. Finally, because the added node is isolated, it does not change the original result under the given customer sequence $T$. As a result, $value(t_{j}|T_{+1})\geq value(t_{j}|T)$ holds.
