# Supplementary material for: The value of leading customers in a crowdfunding-based marketing pattern
Source: PLoS One. 2019 Apr 15;14(4):e0215323. doi: 10.1371/journal.pone.0215323 (PMC6464345; doi:10.1371/journal.pone.0215323)
Supplement: S6 Appendix — (DOCX) [file pone.0215323.s006.docx]

Proof of Property 1.

Without loss of generality, a line network contains *n* customers, and their IDs are 1 to *n*, which also reflects their sequence orders, as displayed in Fig 2. Given $\alpha$ and $\beta$, when a newcomer *n*+1 is added into the line network, we next calculate how much value is increased of the existing *n* leading customers. For any $j\in\{1,2,\cdots,n\}$, we denote $\Delta value(t_{j})$ $=value(t_{j}|T_{+1})-$ $value(t_{j}|T)$. Thus, according to Equation (22) in **Result 3** and the fact that the line network structure can be expressed as a tridiagonal matrix, we can achieve the following relations:

$\Delta value(t_{j})$∝$\beta^{-(n+2-j)}$,

which implies that when $k\in\{2,\cdots,n\}$,

$\frac{\Delta value(t_{k})}{\Delta value(t_{k-1})}$∝$\beta$.

Thus, the two rules hold under the line network structure and the given precondition.
